# Combined use of NK cells and radiotherapy in the treatment of solid tumors

**DOI:** 10.3389/fimmu.2023.1306534

**Published:** 2024-01-09

**Authors:** Wang Zheng, Sunkai Ling, Yuandong Cao, Chunlin Shao, Xinchen Sun

**Affiliations:** ^1^ Department of Radiation Oncology, The First Affiliated Hospital of Nanjing Medical University, Nanjing, China; ^2^ Institution of Radiation Medicine, Shanghai Medical College, Fudan University, Shanghai, China

**Keywords:** radiotherapy, natural killer cell, tumor microenvironment, granzyme B, CGAS/STING signaling

## Abstract

Natural killer (NK) cells are innate lymphocytes possessing potent tumor surveillance and elimination activity. Increasing attention is being focused on the role of NK cells in integral antitumor strategies (especially immunotherapy). Of note, therapeutic efficacy is considerable dependent on two parameters: the infiltration and cytotoxicity of NK cells in tumor microenvironment (TME), both of which are impaired by several obstacles (e.g., chemokines, hypoxia). Strategies to overcome such barriers are needed. Radiotherapy is a conventional modality employed to cure solid tumors. Recent studies suggest that radiotherapy not only damages tumor cells directly, but also enhances tumor recognition by immune cells through altering molecular expression of tumor or immune cells via the *in situ* or abscopal effect. Thus, radiotherapy may rebuild a NK cells-favored TME, and thus provide a cost-effective approach to improve the infiltration of NK cells into solid tumors, as well as elevate immune-activity. Moreover, the radioresistance of tumor always hampers the response to radiotherapy. Noteworthy, the puissant cytotoxic activity of NK cells not only kills tumor cells directly, but also increases the response of tumors to radiation via activating several radiosensitization pathways. Herein, we review the mechanisms by which NK cells and radiotherapy mutually promote their killing function against solid malignancies. We also discuss potential strategies harnessing such features in combined anticancer care.

## 1 Introduction

Adoptive cell therapy (ACT) is an extremely personalized immunotherapy which involves adoptive transfer of autologous lymphocytes into patients with advanced malignancies, especially hematologic neoplasms, who have exhibited remarkable tumor eradication (1). Since 1988, when the first demonstration of autologous tumor-infiltrating T lymphocytes-based ACT mediated objective tumor regression in patients with metastatic melanoma, T lymphocytes were the only authoritative ACT target over almost twenty years (2). Nonetheless, numerous “unsuccessful” cases did remind us T lymphocytes-based ACT was not always as satisfactory as we thought, and rate of relapse or no-remission was significantly higher than we could accept (3–6). Mechanically, these confusions are derived from several barriers in T lymphocytes therapy, including severe life-threatening toxicities, restricted trafficking, limited tumor infiltration, and in particular, modest anti-tumor activity and antigen escape (7–10). Apart from seeking technological improvements for solving these obstacles, researchers also explored other antitumor lymphocytes, which could be chosen for high-avidity tumor recognition, as well as for effective agents required to induce cancer elimination in the use of ACT. The natural killer (NK) cell-based immunotherapy was then developed.

As a subset of innate lymphoid cells, NK cells are currently considered exerting natural cytotoxicity against primary malignancy and metastasis by suppressing proliferation, migration and colonization to distant tissues (11). In addition to their cytotoxic role, NK cells have been reported to produce plenty of cytokines, mainly interferon-γ (IFN-γ), to modulate adaptive immune responses or other related pathways (12, 13). Of note, NK cells are capable of distinguishing abnormal cells from healthy ones, providing accurate anticancer cytotoxicity and alleviating off-target complications (14, 15), which should be the more important reason for NK cells naturally emerging as a promising target for ACT. In the first pioneering clinical practice in 2005, Millier and colleagues adoptively transferred allogeneic activated NK cells into patients with acute myeloid leukemia, leading to major tumor regression (16). Up to now, numerous clinical studies revealed that adoptively transferred NK cells triggered robust eradication of primary leukemic blasts, substantially reduced the burden of acute myeloid leukemia, and lengthened the overall survival of patients (17–19). However in solid tumors, the therapeutic efficiency of NK cell-based ACT is much less than desired (20, 21), and results from more than 40 clinical trials have been inconsistent (22). The *de facto* clinical benefits of alloreactive NK cells in patients with neurologic tumors have been documented in two instances (23, 24). In contrast to hematologic malignancies, solid tumors are characterized by dense physical structures and a supportive TME that is often impervious to effector immune cells (25). Thus, two inevitable parameters are reported to mainly determine the therapeutic efficacy for alloreactive NK cells in patients with solid tumors: (i) intra-tumoral infiltration (26); (ii) persistence in a robust anti-tumor state (16). With respect to the determinants against infiltration of NK cells into tumors, C-X-C motif chemokine ligand 9 (CXCL9), CXCL10, or CXCL11 (which are all ligands to the C-X-C motif chemokine receptor 3 (CXCR3), a receptor that plays a crucial part in the recruitment of NK cells in solid tumors) have shown substantial secretion in an experimental model of lymphoma, lung adenocarcinomas, and melanomas tissues (27–29). Several studies have also uncovered the obstacles preventing persistence of an activated state for transferred NK cells, including the abundance of interleukin (IL) -2, IL-12, IL-15 (and many other cytokines) and hypoxia or reactive oxygen species (ROS) in the TME (30–33). Obviously, the two parameters mentioned above are deeply affected by the functional factors in TME. Under normal circumstances, the specificity of the TME tends to prevent the infiltration of NK cells into solid tumors, as well as inhibit anticancer activation. Efforts have been made to remove functional damage to NK cells by pharmaceutical or non-pharmaceutical means (34–36), but it takes years for these external interventions to enter clinical practice.

Over decades, radiotherapy has been a conventional therapeutic modality for patients with solid neoplasms. In recent years, radiotherapy has been employed not only for its direct tumoral-killing ability, but also TME modulation (37–40). An increasingly emphasized byproduct of the radiation damage to tumor cells is the secretion/release of various cytokines, chemokines or tumor-associated antigens (TAAs) in TME (41, 42), contributing to the underlying role of radiotherapy to serve as an “*in situ* vaccine”, rendering immune-insensitive tumors responsive to immunotherapy. Thus, it has been speculated that radiotherapy mediates a favorable TME for the infiltration and activation of NK cells in solid tumors. Additionally, despite survival rates of patients with solid malignancies are elevated by assistance of radiotherapy, radioresistant properties of tumors remain a significant barrier for curative treatment (43). Clinical interferences on traditional radioresistant pathways, including DNA damage repair or protective autophagy, still did not improve therapeutic efficiency to a gratifying level. In the era of cancer immunotherapy, a new appreciation of the role played by immune system in governing the therapeutic effect of radiotherapy has caused a major spike in interest. As a novel and pivotal part of cancer immunotherapy, NK cell-based ACT definitely has tight interaction with radiotherapy, which has obtained considerable support from preclinical and clinical data (**Table 1**). Underlying these trials are the hope that radiotherapy would optimize immune activity of NK cells in solid tumors, while alloreactive NK cells further amplify the tumor response of radiotherapy.

**Table 1:** Clinical trials testing allogenic NK cells therapy combined with radiotherapy in patients with solid tumors.

Indication(s)	Phase	Status	N_0_	Other regimens	Ref.
Gastric cancer Head and neck cancer	2	Recruiting	55	PD-1 blocker	NCT04847466
Ovarian cancer	2	Terminated	14	Cyclophosphamide Fludarabine	NCT00652899
Breast cancer	2	Terminated	6	Cyclophosphamide Fludarabine	NCT00376805
Sarcomas	Not applicable	Recruiting	10	Cyclophosphamide Fludarabine IL-12	NCT05952310
Solid tumors	2	Active, not recruiting	15	Cyclophosphamide Fludarabine	NCT02100891
Glioblastoma	1	Recruiting	5	None	IRCT20170122032121N5
High grade glioma	2	Recruiting	40	Chemotherapy	IRCT20170122032121N7

Source: http://www.clinicaltrials.gov; https://trialsearch.who.int.

Herein, we review the mechanisms by which NK cells and radiotherapy mutually promote their killing function against solid malignancies. We also discuss potential strategies harnessing such features in supporting anticancer care.

## 2 Radiotherapy encourages the optimal anti-tumor activity of NK cells

Several environmental features play essential roles in preventing the promising function of NK cells in solid tumors. Radiotherapy (a robust modulator of the TME) can eliminate such barriers and contributes (but is not limited) to ameliorating the trafficking of NK cells as well as enhanced cytotoxic functions.

### 2.1 Facilitation of trafficking of NK cells

For almost all anticancer chemo- or immune-therapy, the inevitable question in clinical practice is how to enhance the abundance of functional agents intratumorally. NK cell-based ACT has no exception. However, investigation of tumor biopsy specimens for various solid neoplasms, no matter patients derived or animal derived, have demonstrated little infiltration of NK cells into these tumors (44–46). The interactions between chemokines and their corresponding chemokine receptors have been widely explored for their roles in driving NK cells trafficking (47, 48). Chemokine receptors are grouped by structure into four types: C-X-C chemokine receptor (CXCR), C-C chemokine receptor (CCR), C-X3-C chemokine receptor (CX3CR), and XCR (49), among which CXCR3 expression is to date one of the marker providing the most reliable message about the accumulation of NK cells. In general, CXCR3 is highly expressed on the surface of NK cells, and it drives NK cell-specific chemotaxis toward the CXCR3 ligands CXCL9, CXCL10 and CXCL11, which are secreted by tumor cells into TME and usually expressed at relative low levels in homeostatic condition (50–52). Hence, strategies to increase CXCR3 expression by NK cells and/or raise the abundance of CXCR3 ligands secreted in the TME may lead to enhanced recruitment of NK cells into solid malignancies. Cytokine stimulation upon NK cells was proved to be able to modulate the expression of CXCR3. It was shown that *ex vivo* expansion of NK cells in the coculture of IL-2 and feeder cells (EBV-LCLs) significantly upregulated the expression of CXCR3 on NK cells, resulting in increased migratory capacity toward CXCL10-producing tumor cells *in vitro* (27). Besides, pharmaceutical interventions such as BXCL701 (inhibitor of dipeptidyl peptidase) and tazemetostat (a polycomb histone-lysine N-methyltransferase enzyme-EZH2 inhibitor) can stimulate CXCL9/10 production, which has been shown to lead to enhanced NK-cell infiltration as well as cytotoxicity and eradication of pancreatic ductal adenocarcinoma in a mouse model (53, 54). Radiotherapy has been reported to elicit similar results as those reported above in several experimental and clinical models. Specifically, radiotherapy combined with inhibitors of ATR serine/threonine kinase triggered CXCL10 expression and increased infiltration of innate immune cells (including NK cells) in models of human papillomavirus-driven malignancies (55). Also, in models of Kirsten rat sarcoma virus-mutated carcinomas, radiotherapy enhanced CXCL10 secretion, promoted anticancer immunity, and prolonged survival (56). Furthermore, the mechanisms by which radiotherapy influences expression of CXCR3 ligands have been postulated. Recent research has suggested that radiotherapy drives a type-I IFN response that culminates with CXCL10 secretion via accumulation of nuclear and mitochondrial DNA in the cytoplasm and consequent activation of cyclic guanosine monophosphate-adenosine monophosphate synthase (cGAS) (57). Moreover, radiotherapy can also lead to phosphorylation of p38 mitogen-activated protein kinase and increased expression of signal transducer and activator of transcription 1 (STAT1) which, ultimately, augments CXCL10 production (58, 59).

Apart from driving infiltration of NK cells into solid tumors, CXCR3 is also preferentially expressed on a number of cancer cells. In addition, several evidences reveal that CXCR3 on tumor cells surface facilitates the proliferation and metastasis of a variety of malignancies, including colon cancer (60) and melanoma (61), and CXCR3 blockade has shown a prospective anti-metastatic potential in breast cancer (62). Mechanically, the pairing of tumoral CXCR3 and its ligands may activate downstream Ras/ERK and PI3K/AKT pathways (63, 64). Thus, the relationship between CXCL9, CXCL10, CXCL11/CXCR3 axis and tumor development or patient prognosis is still controversial. In some cases, consensus is made upon the association between CXCR3 or its ligands expression and negative response to existing treatments (65, 66), while other reports reveal an opposite result (67–69). The theoretical base is still under study. At least, these results encourage us that patients with certain types of malignancies may benefit from radiotherapy-induced NK cells intratumoral infiltration, while not suffer from increased metastasis. Whereas, it also reminds us when combined NK cell-based ACT and radiotherapy in solid tumor treatment, we should detect CXCR3 and its ligands expression in cancer cells, and pathways (as Ras/ERK or PI3K/AKT) inhibitors should be introduced to prevent tumor metastasis in time. According to the interesting work by Liu WS and colleagues (70), strategy which systematically establishes a score utilizing CXCR3 and its ligands abundances in TME to predict tumor metastasis during NK cell-based ACT and radiotherapy combined therapy may be beneficial and promising.

Apart from the essential CXCR3, additional chemokine receptors, including CXCR4, CCR7 and CX3CR, have been reported to promote the recruitment of NK cells to the TME of solid neoplasms (71–74). Importantly, with no pharmaceutical strategies clinically available, radiotherapy seems to be the most cost-effective strategy to drive the expression of these chemokine receptors or their ligands. Specifically, expression of CXCR4 and its ligand CXCL12 were found to be increased after radiotherapy in patients with metastatic prostate cancer (75), rectal cancer (76), or nasopharyngeal carcinoma (77). When exploring tumor-infiltrating lymphocytes (TILs) after radiotherapy in patients with prostate cancer, researchers observed a higher number of CCR7^+^ TILs in tumor biopsies post-radiotherapy (78). In an *ex vivo* experiment, single-dose radiation promoted the migration of CX3CR1^+^ immune cells markedly and upregulated expression of its cognate ligand CX3CL1 (79, 80). *In vivo* and *in vitro* data have shown the positive regulation of radiotherapy on expression of CXCR4, CCR7, or CX3CR1, but the fundamental basis has yet to be identified.

However, similar with CXCR3, CXCR4 is not a NK cell-specific chemokine receptor either. CXCR4 expression is also abundant in immunosuppressive cells such as myeloid-derived suppressor cells (MDSCs). A recent study demonstrated that radiotherapy induced infiltration of CXCR4^+^ MDSCs into the microenvironment of glioblastoma, and led to diminished anti-tumor immunity (81). A similar contradiction can also be found via a mechanism involving CXCL8 secretion. The mammalian target of rapamycin (mTOR)-p65 axis showed sustained activation and was responsible for radiotherapy-induced CXCL8 release, which caused directional migration of NK cells to the TME in patients with pancreatic cancer (82). However, it has also been reported that expression of squamous cell carcinoma antigen 1 (clinical biomarker of a poor response to anticancer therapy) resulted in increased CXCL8 expression along with promotion of intra-tumoral trafficking of MDSCs in response to radiotherapy (83). Thus, combination therapy, such as radiotherapy plus SX-682 (CXCR1/2 inhibitor) (84), could abrogate the recruitment of tumor MDSCs and enhance the tumor infiltration and therapeutic efficacy of NK cells.

The data stated above reveal that trafficking of NK cells into solid tumors can be ameliorated by radiotherapy through driving abundant chemokines receptors expression on NK cells or corresponding ligands secretion by tumor cells into TME that favor NK-cell infiltration.

### 2.2 Promoting the cytotoxicity of NK cells

The cytotoxicity of NK cells involves secretion of cytotoxic granules containing the pore-forming protein perforin and procaspase-cleaving granzyme B (GzmB), which induces apoptosis in targeted tumor cells (85). Also, NK cells can trigger target-cell apoptosis via the death receptors tumor necrosis factor (TNF)-related apoptosis-inducing ligand (TRAIL) and FAS ligand (86). Unfortunately, no report has shown a positive correlation between radiotherapy and secretion of perforin or GzmB by NK cells. However, numerous works have suggested a link between radiotherapy and expression/secretion of GzmB by cluster of differentiation (CD)8^+^ T cells (whose major cytotoxic executors are also GzmB and perforin) in models of prostate cancer (87), melanoma (88), or hepatocellular carcinoma (89). An *ex vivo* study in a model of colorectal cancer revealed that a radiotherapy-induced cGAS signaling pathway may favor GzmB expression by CD8^+^ T cells (90). A case report elucidated that upregulated expression of perforin in T cells was due to radiotherapy in a patient with metastatic gastric cancer (especially in the disease-resolution period) (91). Even though the synthesis, mobilization, and secretion of GzmB and perforin involve complex signal-transduction pathways, similar mechanisms are observed in T cells and NK cells (92–94). Therefore, it is reasonable to speculate that radiotherapy should be a promising strategy to stimulate secretion of GzmB and perforin in the TME by NK cells for solid tumors. However, direct evidence is needed to define this link.

Besides cytotoxic granules, NK cells also exert antitumor effects through secretion of cytokines such as TRAIL. The latter induces the apoptosis of cancer cells directly by bonding to its death receptors (DR4/DR5) (95). However, almost all tumors are not sensitive to TRAIL, including astrocytoma, chronic lymphocytic leukemia, medulloblastoma, and meningioma (96). The cause of this lack of sensitivity in erythroleukemia was investigated by Di Pietro and colleagues: the low surface expression of the TRAIL receptor DR4 was suggested to be the reason. Furthermore, they concluded that a low (1.5 Gy) or high (15 Gy) single dose of radiation could sensitize erythroleukemic cells to TRAIL mediated-cytotoxicity by selective upregulation of TRAIL-R1 (DR4) expression (97). A similar result was also observed in a mouse model of TRAIL-R2 (DR5) deficiency designed by Niklas and colleagues, whose study put radiotherapy “center stage” in TRAIL-induced organ-specific damage (98). Hence, we wonder whether radiotherapy could increase the response to TRAIL-induced apoptosis in other tumors and, ultimately, boost the cytotoxicity of NK cells. Apart from increasing expression of the death receptors of TRAIL, radiotherapy (a genotoxic agent) may sensitize cancer cells to TRAIL by activating diverse TNF-associated apoptotic pathways in tumor cells, such as caspase-8, p38, or nuclear factor-kappa B (99–101).

The degree to which NK cells are active against tumor cells is based on the activation state of NK cells, which is regulated in large part by cytokines (e.g., IL-12, IL-18, type-I IFN) derived from other immune cells, including dendritic cells (DCs) and monocytes (102–104). Interestingly, preclinical studies have been conducted over decades combining DCs-based immunotherapy with radiotherapy, and clinical benefit (at least a partial response) was exhibited when compared with administration of DCs alone. In a mouse-bearing sarcoma model, radiotherapy plus DCs treatment resulted in favorable infiltration of DCs into the TME to aid tumor eradication (105). However, in an *ex vivo* co-culture experiment, a significant difference in apoptosis or necrosis between DCs-administration alone and DCs plus radiotherapy was not detected in cancer cells. Explanation was not given in that study, but we hypothesize that radiotherapy promoted the antitumor effect of DCs via crosstalk between DCs and other immune-active cells (e.g., NK cells, CD8^+^ T cells) rather that DCs. Another study using models of pancreatic carcinoma and colorectal carcinoma showed that radiotherapy facilitated DCs maturation, followed by enhanced production of type-I IFN, and contributed to the activation of CD8^+^ T cells (106). Undoubtedly, NK cells could be activated simultaneously by type-I IFN also. To ascertain how radiotherapy activates DCs, researchers suggested that the cGAS-stimulator of interferon genes (STING; an endoplasmic reticulum-associated protein) axis was indispensable in radiation-induced production of type-I IFN by DCs. DNA fragments from irradiated cancer cells gained access to a cytosolic DNA-sensing pathway in DCs via direct cell–cell contact to trigger induction of STING-dependent type-I IFN, which further drove the adaptive immune response to radiation (107). The cGAS-STING pathway plays an essential part in expression of the NK-cell trafficking-associated ligand CXCL10 by irradiated tumor cells but also in the production of type-I IFN in DCs. Further studies may reveal more information about the cGAS-STING axis in radiotherapy-mediated activation of NK cells.

Activation of NK cells is also reliant on tumor-associated antigens (TAAs) expressed by cancer cells. TAAs are antigenic proteins produced in tumor cells, which can trigger an autoantibody response in patients (108). Numerous studies have reported that TAAs markedly influence the immunosurveillance of NK cells for aberrant cells or tumor cells, and partly determine the clinical benefits of adaptively transferred NK cells (109–112). The TAAs, New York esophageal squamous cell carcinoma-1 (NY-ESO-1), CD20, and CD48 are the most reported in terms of NK-cell stimulation. In recent years, radiotherapy-mediated genomic alteration of TAAs has attracted attention. In a single-arm feasibility study, patients with metastatic renal cell carcinoma were treated with stereotactic body radiotherapy (SBRT; 15 Gy) at the primary lesion in a single fraction, and specimens were analyzed for TAA expression at 4 weeks (113). Radiotherapy-treated tumors had enhanced expression of several TAAs, including carbonic anhydrase IX (CA9), trophoblast glycoprotein (TPBG), NY-ESO-1, and mucin-1. Interestingly, NY-ESO-1 seems to be the only intersection between radiotherapy-induced change in TAAs and NK cell stimulation-associated TAAs. Inspiringly, in a patient diagnosed with locally advanced unresectable gastric cancer, radiotherapy led to upregulation of NY-ESO-1 expression in the tumor, and enhanced the anticancer efficacy of anti-programmed cell death protein 1-based immunotherapy that correlated with a beneficial clinical outcome (91). Direct evidence identifying a pronounced relationship between radiotherapy, TAAs (e.g., NY-ESO-1), and NK-cell stimulation is lacking, but the above case report by Merhi M and colleagues suggests that radiotherapy may activate the NK-cell response to malignancies through upregulating expression of NY-ESO-1 or other TAAs.

Several metabolic features in the TME have been found to exert a considerable negative impact upon NK-cell activation. The rapid and uncontrolled proliferation of tumors leads to an insufficient blood supply and limits oxygen availability, so hypoxia is the most prominent metabolism-associated TME feature in nearly all solid tumors (114). Studies have reported that under hypoxia, there is a modulation of the genes associated with immunomodulatory effects and reduction in the number of activating receptors (e.g., NKG2D, NKp46), which result in impaired effector functions in NK cells (115–117). Hypoxia-inducible factor (HIF)-1α is considered to be responsible for this genetic regulation (though the specific pathway has yet to be uncovered). Reversing hypoxia is a promising method to reactivate NK cells, so radiotherapy has attracted attention. As one of the “4R” principles post-irradiation, reoxygenation occurs intratumorally in the early phase of radiotherapy. In 20 patients with head and neck cancer who received intensity-modulated radiotherapy (70 Gy/35 fractions), the intensity and volume of tumor hypoxia declined rapidly, and ^18^F-fluorodeoxyglucose-positron emission tomography suggested gradually decreased fluorodeoxyglucose uptake during intensity-modulated radiotherapy, which indicated a substantial tumoricidal effect over the entire treatment (118). Notably, a recent study demonstrated radiotherapy-induced tumoral reoxygenation to be dose-dependent. In six patients with lung cancer undergoing SBRT (18 Gy/fraction), increased (or at least persistent) tumor hypoxia was observed (119). In a prospective study of lung cancer, SBRT (12-13 Gy/fraction) caused a considerable reduction in tumor hypoxia and a favorable prognosis (120). Thus, at least in lung cancer, a high dose of radiation (18 Gy/fraction) should be the upper limit for radiotherapy-induced reoxygenation. Besides, tumor specificity may also be taken into consideration because a high dose (20 Gy) in patients with prostate cancer led to a reduced hypoxic volume in nearly all patients (121). In summary, using radiotherapy to eliminate hypoxia in solid tumors and thereby improve NK-cell cytotoxicity is a feasible and promising strategy. However, the radiation dose and tumor specificity must be evaluated individually. In addition, whether HIF-1α has a vital role in this regulation is not known.

**Figure 1:**
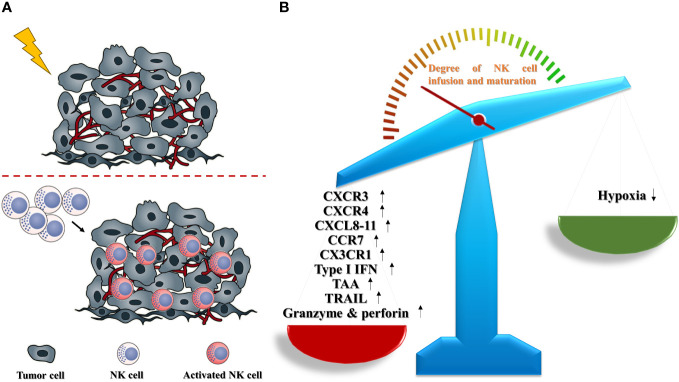
Radiotherapy promotes the infiltration and activation of NK cells in solid tumors (schematic). **(A)** In solid tumors, radiotherapy contributes to a boost trafficking of NK cells into TME, and accelerates the functional maturation of infused NK cells. **(B)** Radiotherapy could remodel the TME to make a NK-favored microenvironment via upregulating NK-promoted parameters including CXCR3, CXCR4, CXCL8-11, CCR7, CX3CR1, type I IFN, TAA, TRAIL, granzyme and perforin, as well as abating NK-suppressed obstacle, especially hypoxia.

The evidence stated above suggests that fractionated radiotherapy is a promising strategy for the recruitment and cytotoxic function of NK cells in solid tumors because it can eliminate inhibitory signals and augment enhancement signals simultaneously (**Figure 1**).

## 3 NK cells amplify radiotherapy-induced anticancer damage

Radiotherapy has been applied against almost all types of solid tumors for decades. The efficacy of radiotherapy in curing some malignancies can be suboptimal. The radioresistance of these tumors can be the reason for this poor response to radiotherapy. Various signaling pathways, such as DNA damage response (122, 123) and autophagic apoptosis (124, 125) are involved in the radioresistance of tumors. Most radiosensitizers targeting these mechanisms have been under preclinical study for years, but few have been approved for clinical use. During mechanistic studies, researchers observed a link between microenvironmental NK cells and tumor radioresistance. In general, NK cells can improve the tumor response to radiotherapy via two pathways: (i) cytokines (or cytotoxic granules) secretion (2); molecular signal transduction.

### 3.1 Secretion of cytokines or cytotoxic granules enhance tumor radiosensitivity

Stimulated NK cells can produce cytotoxic granules (e.g., perforin, granzymes) as well as multiple cytokines (e.g., IFN-γ, TNF-α, IL-13) (14, 126). NK cells kill tumor cells directly or increase the immunosurveillance of other immune cells (e.g., CD8^+^ T cells) indirectly via secretion of these effectors, but biological processes in cancer cells (e.g., ferroptosis, autophagy, repair of DNA damage) may also be influenced by them, which will influence the radioresistance of tumors.

Among NK cell-derived cytokines, IFN-γ is the most fundamental factor involved in the crosstalk of NK cells with almost all immune-active cells, such as CD8^+^ T cells, helper CD4^+^ T cells, antigen-presenting cells, and macrophages (127). The effects of IFN-γ on various biological processes related to radiosensitization in tumor cells are beginning to be identified. In a model of prostate cancer in mice, NK cells secreting IFN-γ increased ferroptosis in cancer cells which, in turn, enhanced NK-cell function (including IFN-γ production) (128). Peng and colleagues suggested IFN-γ signaling to be a natural ferroptosis-promoting mechanism in tumors involving the metabolism of C16 and C18 acyl chain-containing phospholipids (129). Both of those works did not study the continuous impact on the radioresistance of tumors, but the role of enhanced ferroptosis in sensitizing various types of neoplasm to radiation is accepted. Numerous preclinical studies have disclosed a relatively low level of ferroptosis in malignancies with a weak response to radiotherapy, and that pharmaceutical induction of ferroptosis resensitized these tumors to radiation significantly (130–132). Hence, by triggering ferroptosis, IFN-γ should overcome the radioresistance of tumors and enhance radiation-induced damage. Rao and colleagues suggested that all-trans retinoic acid can overwhelm the radioresistance of solid tumors by inducing inflammatory macrophage-derived IFN-γ (133). Hence, apart from ferroptosis or fatty-acid metabolism, more pathways may participate in the IFN-γ-mediated radiosensitization of solid tumors.

TNF-α is synthesized and secreted mainly by NK cells. In common with other TNF family members, TNF-α is involved in the maintenance and homeostasis of the immune system, inflammation, and defense against malignancy (134). Apart from these immunosurveillance functions, a unique influence of TNF-α upon radiosensitization in solid tumors was reported decades earlier. In a model of prostate cancer, when cells were irradiated 24 h after exposure to TNF-α, increased cell death was observed. In contrast, radiation delivered 24 h before TNF-α exposure did not lead to more cell death than after TNF-α alone (135). This finding suggested that TNF-α sensitized tumors to radiotherapy. A similar result was observed in a model of B-cell lymphoma treated with radiation plus ceramide inhibition (136). Kimura and colleagues believed that TNF-α sensitized tumors to radiation by increasing intracellular ceramide production.

IL-13 is a pleiotropic immunoregulatory cytokine produced by Th2 cells, NK cells, or other cells of the innate immune system (137). In an *ex vivo* experiment, isorhamnetin treatment increased the radiosensitivity of non-small-cell lung cancer cells, and genetic knockdown of IL-13 expression eliminated isorhamnetin-mediated radiosensitivity in cells (138). This result reflected a role of IL-13 in overcoming resistance to radiotherapy in solid tumors. In another study, “protumor” M2-polarized macrophages in the microenvironment of breast cancer were augmented after IL-13 administration, which further mediated tumor radioresistance; however, inhibition of IL-13-mediated M2 polarization of macrophages by PM37 could prevent radioresistance (139). This contradictory IL-13-mediated removal or maintenance of radioresistance may be derived from the difference between *in vitro* and *in vivo* experimental models. In a simple model of cancer cells, IL-13-related pathways can sensitize cells to radiation without consideration of the effects on other types of immune cell but, in tumor-bearing mouse model or humans, this effect is inevitable. Accordingly, if taking advantage of IL-13 derived from mature NK cells for reducing tumor radioresistance, the ability of IL-13 to promote groups of protumor immune cells must be considered.

One mechanism of NK cell-mediated tumor killing involves granzymes that fragment nuclear DNA and leads to cell death (85). The biologic/pathologic functions of most granzymes are not known, but GzmB contributes to DNA fragmentation and results in cell death directly. However, studies on GzmB-induced radiosensitization in lung cancer and prostate cancer, respectively, are interesting. Researchers found that administration of resveratrol or AuNPs-si-SP1 could increase radiosensitivity markedly by upregulating the expression and synthesis of GzmB in cancer cells (140, 141). GzmB secreted by NK cells or other immune cells and GzmB synthesized by tumor cells are not identical. Those two studies were the first to focus on the regulatory role of GzmB on tumor radioresistance rather than its direct apoptosis-inducing ability, which expand our knowledge on the biological functions of GzmB.

### 3.2 Molecules from NK cell-derived exosomes activate radiosensitization pathways

Exosomes are nanovesicles secreted actively by almost all cell types. Exosomes deliver certain intracellular molecules (e.g., nucleic acids, proteins, lipids) to target cells (142). Studies have focused mainly on exosomes released from cancer cells, but the functions and characteristics of exosomes derived from NK cells have been explored in recent years. Reports have suggested that NK cell-derived exosomes could be assimilated by tumors in a time-dependent manner, and such uptake improved the response to anti-cancer treatments such as cisplatin in melanoma and ovarian carcinoma (143, 144). The mechanisms by which NK cell-released exosomes exert this sensitization function have not been elucidated. However, small RNA-sequencing of exosomes secreted by NK cells identified a specific *repertoire* of NK exosome-associated microRNAs (mIRs) (145). Interestingly, miR-10b-5p (146), miR-92a-3p (147), miR-146a-5p (148), and miR-99a-5p (149) specifically abated tumor radioresistance involving multiple molecular pathways such as ATM/ATR serine/threonine kinase, Janus Kinase, and protein kinase B/mTOR. Apparently, miRNA transduction into tumor cells by NK-derived exosomes may increase the radiosensitivity of multiple malignancy types.

**Figure 2:**
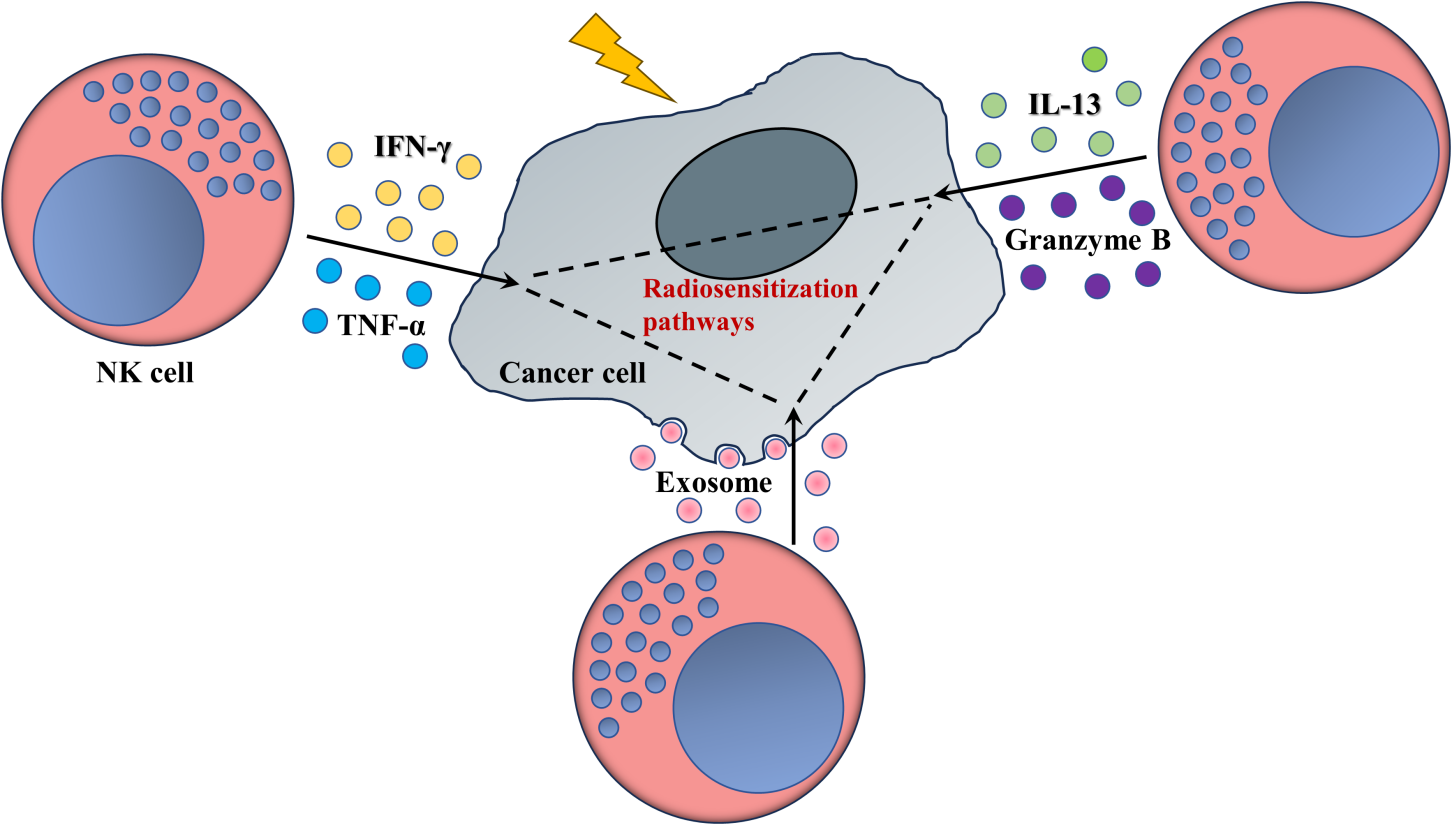
NK cells enhance the response of tumors to radiotherapy (schematic). Mature NK cells secrete various cytokines or cytotoxic granules, as well as plenty of exosomes, whose interactions with radiosensitivity-related pathways or biological process, as ferroptosis, fatty acid metabolism or miRNAs networks, access valid ability in pruning radioresistance of cancer cells.

In summary, the data stated above suggest an indirect sensitization role rather than a direct death-inducing function of NK cell-derived cytotoxic granules, cytokines or exosomes in anti-tumoral radiotherapy (**Figure 2**).

## 4 Concluding remarks and future perspectives

NK cells have been suggested to be candidates for anti-tumor therapy in recent years, and inspiring data have been documented in individuals with hematologic neoplasms. Nevertheless, application of NK cells in the treatment of solid malignancies is hampered by many obstacles (150, 151). Despite strategies aimed at promoting the infiltration and activation of NK cells in the TME, translation into clinical practice is difficult. In contrast, priming the TME with radiotherapy could “jumpstart” the immune activity of NK cells by providing a permissive microenvironment for them. Moreover, mature NK cells have been reported to improve the response of solid tumors to radiotherapy. This complementary function between radiotherapy and NK cells likely results in a synergistic effect on tumor eradication, which is why a combination of radiotherapy and NK-based ACT is crucial against cancer. This strategy should be valid but direct evidence to prove its efficacy and safety is lacking. We expect more clinical trials focusing on radiotherapy and NK-based ACT but the histology subtype, radiotherapy dose/sequence, and interference by other immune cells in the TME must be taken into consideration.

In a whole, radiotherapy could be one of the keys to unlock the potential of therapy based on the transfer of NK cells against cancer, and conversely, infusion and maturation of NK cells may resolve the long-standing radioresistance in solid tumors.

## Author contributions

WZ: Writing – original draft. SL: Investigation, Writing – review & editing. YC: Data curation, Writing – review & editing. CS: Writing – review & editing. XS: Writing – review & editing.
